# FAIR-Net: A Fuzzy Autoencoder and Interpretable Rule-Based Network for Ancient Chinese Character Recognition

**DOI:** 10.3390/s25185928

**Published:** 2025-09-22

**Authors:** Yanling Ge, Yunmeng Zhang, Seok-Beom Roh

**Affiliations:** 1 School of Information Science and Engineering, Linyi University, Linyi 276000, China; geyanling@lyu.edu.cn; 2 School of Chinese Language and Literature, Shandong Normal University, Jinan 250014, China; 3 Department of Electronics and Computer Engineering, Seokyeong University, Seoul 136-704, Republic of Korea

**Keywords:** ancient character recognition, fuzzy neural networks, Chinese script digitization, Fuzzy C-Means clustering, Iteratively Reweighted Least Squares Estimation

## Abstract

**Highlights:**

**What are the main findings?**

- Developed **FAIR-Net**, a hybrid model combining deep autoencoder-based feature extraction with an interpretable fuzzy rule-based classifier for ancient Chinese character recognition.
- Achieved **state-of-the-art accuracy and high efficiency**, with 97.91% accuracy on modern handwritten datasets and 83.25% on a 9233-class ancient character dataset, while being 5.5× faster than SwinT-v2-small.

**What is the implication of the main finding?**

- Demonstrates that integrating interpretable fuzzy rules with deep representations can **significantly enhance both performance and interpretability** in large-scale character recognition tasks.
- Provides a **practical and explainable solution** for processing degraded and stylistically diverse ancient scripts, enabling future applications in digital humanities and cultural heritage preservation.

**Abstract:**

Ancient Chinese scripts—including oracle bone carvings, bronze inscriptions, stone steles, Dunhuang scrolls, and bamboo slips—are rich in historical value but often degraded due to centuries of erosion, damage, and stylistic variability. These issues severely hinder manual transcription and render conventional OCR techniques inadequate, as they are typically trained on modern printed or handwritten text and lack interpretability. To tackle these challenges, we propose FAIR-Net, a hybrid architecture that combines the unsupervised feature learning capacity of a deep autoencoder with the semantic transparency of a fuzzy rule-based classifier. In FAIR-Net, the deep autoencoder first compresses high-resolution character images into low-dimensional, noise-robust embeddings. These embeddings are then passed into a Fuzzy Neural Network (FNN), whose hidden layer leverages Fuzzy C-Means (FCM) clustering to model soft membership degrees and generate human-readable fuzzy rules. The output layer uses Iteratively Reweighted Least Squares Estimation (IRLSE) combined with a Softmax function to produce probabilistic predictions, with all weights constrained as linear mappings to maintain model transparency. We evaluate FAIR-Net on CASIA-HWDB1.0, HWDB1.1, and ICDAR 2013 CompetitionDB, where it achieves a recognition accuracy of 97.91%, significantly outperforming baseline CNNs (*p* < 0.01, Cohen’s d > 0.8) while maintaining the tightest confidence interval (96.88–98.94%) and lowest standard deviation (±1.03%). Additionally, FAIR-Net reduces inference time to 25 s, improving processing efficiency by 41.9% over AlexNet and up to 98.9% over CNN-Fujitsu, while preserving >97.5% accuracy across evaluations. To further assess generalization to historical scripts, FAIR-Net was tested on the Ancient Chinese Character Dataset (9233 classes; 979,907 images), achieving 83.25% accuracy—slightly higher than ResNet101 but 2.49% lower than SwinT-v2-small—while reducing training time by over 5.5× compared to transformer-based baselines. Fuzzy rule visualization confirms enhanced robustness to glyph ambiguities and erosion. Overall, FAIR-Net provides a practical, interpretable, and highly efficient solution for the digitization and preservation of ancient Chinese character corpora.

## 1. Introduction

The digitization of ancient Chinese scripts—including oracle bone carvings, bronze inscriptions, stone steles, Dunhuang scrolls, and bamboo or silk manuscripts—is of paramount importance to cultural preservation, digital humanities, and language heritage restoration [1,2]. These scripts, however, often suffer from centuries of physical degradation, glyph variability, and stylistic inconsistencies [3], which introduce severe challenges for both manual transcription and modern optical character recognition (OCR) systems. Manual efforts are labor-intensive and error-prone [4], while conventional OCR models—typically designed for clean, modern characters—lack the adaptability and semantic reasoning required for recognizing historical scripts with eroded strokes and non-standard structures [5,6].

**Figure 1:**
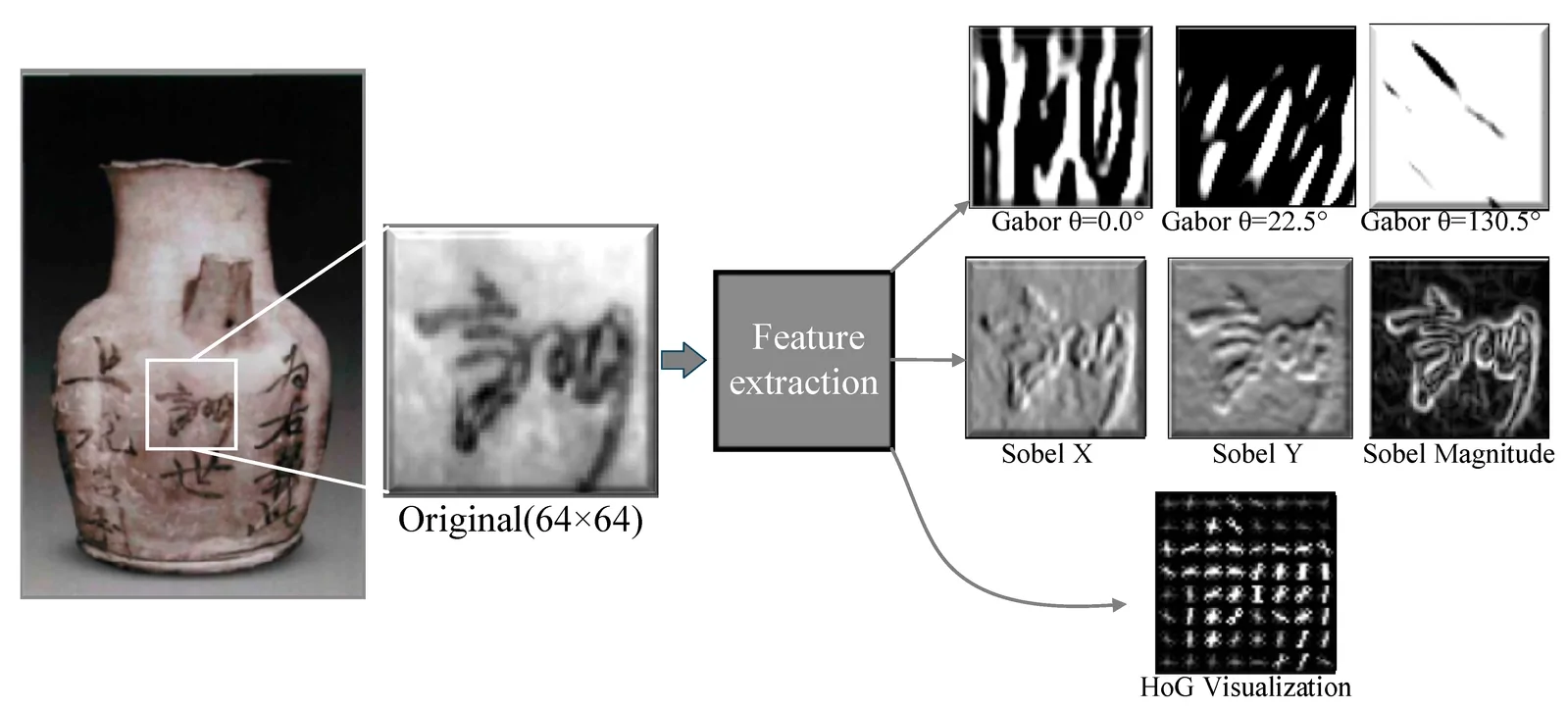
Visualization of Gabor, Sobel gradient, and HoG feature maps extracted from the offline handwritten Chinese character.

As shown in Figure 1, traditional feature extraction techniques, such as Gabor filters [7], Sobel gradients [8], and Histogram of Oriented Gradients (HoG) [9,10], offer interpretable low-level descriptors but fall short in capturing high-level semantic structures in damaged or stylized characters. On the other hand, deep learokning models such as CNNs and RNNs have demonstrated high performance in modern handwriting tasks but are often regarded as black-box systems with limited transparency [11] and high dependency on large annotated corpora—an impractical requirement in the context of historical character collections [12,13].

In recent years, modern Chinese character recognition has increasingly leveraged advanced sequence modeling paradigms such as convolutional recurrent neural networks (CRNN), attention-based encoder–decoder architectures, and transformer backbones. These approaches achieve state-of-the-art performance in scene-text OCR by modeling sequential dependencies among character strokes or text lines [14,15]. However, their strengths rely heavily on large-scale corpora and clear contextual cues, conditions that are not satisfied in the case of ancient scripts. Ancient character recognition is inherently a single-character classification task, where contextual sequence modeling offers limited benefit and where excessive model complexity can lead to overfitting on scarce, imbalanced data. This gap highlights the necessity of developing models that balance robustness, efficiency, and interpretability, rather than simply adopting black-box sequence frameworks [16,17].

To overcome these limitations, we propose FAIR-Net (Fuzzy Autoencoder and Interpretable Rule-based Network), a two-stage hybrid model specifically designed for robust and explainable recognition of ancient Chinese characters. In the first stage, a deep autoencoder performs unsupervised feature extraction, compressing high-resolution character images into compact latent representations that suppress background noise and preserve semantic structure [18,19,20,21,22,23]. Unlike traditional dimensionality reduction methods such as PCA, which lack adaptive feature learning, the autoencoder is well-suited for large-scale, heterogeneous data due to its nonlinear mapping capacity and data-driven learning [24,25,26].

In the second stage, the encoded features are fed into a Fuzzy Neural Network (FNN). This component utilizes Fuzzy C-Means (FCM) clustering to infer soft membership degrees and construct interpretable fuzzy rules, which serve as the hidden layer. Classification is performed through an Iteratively Reweighted Least Squares Estimation (IRLSE) mechanism, integrated with CE loss and L2 regularization to ensure robustness against noisy samples and overfitting. All parameters are constrained as linear mappings, enabling full interpretability and rule traceability.

We validate the effectiveness of FAIR-Net on three benchmark datasets: CASIA-HWDB1.0, CASIA-HWDB1.1, and ICDAR2013 CompetitionDB. The proposed model achieves a peak testing accuracy of 97.91%, outperforming all baselines including GoogLeNet and R-CNN Voting, while maintaining real-time inference efficiency (25 s), as supported by statistical tests and Pareto frontier analysis. Furthermore, ablation studies confirm the necessity of each component (IRLS, CE, L2), revealing their complementary roles in enhancing generalization and semantic consistency. Beyond modern handwritten characters, FAIR-Net was further evaluated on a large-scale Ancient Chinese Character Dataset comprising over 9000 classes across multiple script types (oracle bone, bronze, seal, coin, bamboo/silk, stone inscriptions). The results demonstrate that FAIR-Net retains competitive accuracy while offering substantial training efficiency gains over deep CNN and transformer baselines, and that its fuzzy rule layer provides interpretable decision logic even under extreme class imbalance and stylistic diversity.

In summary, the contributions of this paper are as follows:

(a) We propose FAIR-Net, a novel hybrid framework that combines deep autoencoding with fuzzy rule-based reasoning for robust, interpretable recognition of ancient characters.
(b) We design a fully explainable fuzzy neural network, where each hidden node corresponds to a meaningful fuzzy rule validated through semantic traceability.
(c) We conduct extensive experiments on three public datasets, demonstrating FAIR-Net’s superior performance in both accuracy and efficiency, supported by statistical significance (*p* < 0.01) and large effect sizes (Cohen’s d > 0.8).
(d) We provide visual and quantitative insights into the model’s generalization ability, showing resilience to degraded inputs and consistent rule activation behavior.

The remainder of this paper is organized as follows. Section 2 presents the dimensionality reduction strategy and unsupervised feature extraction process based on a deep autoencoder, designed to preserve semantic structure while suppressing noise. Section 3 describes the architecture and learning mechanism of the proposed fuzzy neural network, including the integration of Fuzzy C-Means clustering, rule construction, and interpretable optimization. Section 4 outlines the complete design of the FAIR-Net framework, emphasizing its modular structure and interpretability features. Section 5 provides a comprehensive evaluation of the model through quantitative experiments, statistical significance testing, efficiency analysis, and ablation studies. Finally, Section 6 concludes the paper with key findings and discusses potential directions for future research.

## 2. Dimensionality Reduction and Feature Extraction via Autoencoder

To efficiently capture semantic structure while suppressing noise and redundancy in large-scale, degraded ancient character images, we adopt a deep autoencoder as the first stage of the proposed FAIR-Net. Unlike traditional dimensionality reduction techniques such as PCA, which rely on linear projections and are sensitive to noise, the autoencoder provides a nonlinear, data-adaptive encoding mechanism that scales effectively to complex, high-variance datasets [27]. Given the considerable diversity and granularity in ancient scripts—spanning thousands of classes with stylistic heterogeneity—PCA fails to preserve class-discriminative structures critical for robust recognition. In contrast, a deep autoencoder can learn hierarchical, high-level features that are both compact and semantically meaningful [28].

We train the autoencoder on grayscale character images of size 64 × 64, normalized to [0, 1]. The encoder consists of three convolutional layers followed by two fully connected layers, directly reducing the input to a configurable latent dimension *d* ∈ {10, 20, 30, 40}. The decoder mirrors this structure in reverse to reconstruct the original image from the same d-dimensional representation. All hidden layers use ReLU, and the output layer uses Sigmoid to match normalized pixel values [25,29].

Once trained, the encoder serves as a fixed feature extractor, mapping input images into compact latent vectors of configurable dimension d. These embeddings retain >90% structural information while reducing computational cost, forming the semantic backbone for the fuzzy rule-based classifier. Algorithm 1 illustrates the procedure of Autoencoder-Based Dimensionality Reduction and Feature Extraction.
**Algorithm 1.** Autoencoder-Based Dimensionality Reduction and Feature ExtractionInput: Preprocessed dataset D = {*x*_i_}, *x*_i_ ∈ ℝ^64×64^ Output: Encoder function *f_en_c(·)* mapping *x* → *z* ∈ ℝ^d^, where *d* ∈ {10, 20, 30, 40} 1  **Initialize** Autoencoder AE:    Encoder: ConvLayer × 3 → FC(4096→512) → FC(512→*d*)    Decoder: FC(*d* → 512) → FC(512→4096) → DeconvLayer × 3    Activation: ReLU (hidden), Sigmoid (output)    Loss function: L = MSE(*x*, AE(*x*))    Optimizer: Adam (learning rate η = 1 × 10^−3^) 2  **for** epoch = 1 to 50 **do** 3    **for each** mini-batch ℬ ⊂ 𝒟, |ℬ| = 128 **do** 4      $\hat{z}$ ← *f_en_c*(B)
5      $\hat{x}$ ← *fd_e_c*($\hat{z}$) 6      Compute loss: L ← MSE(B$,\;\hat{x}$)
7      Backpropagate ∇L and update AE parameters 8    **end for** 9    **if** validation loss not improved for 5 epochs **then** 10      break 11    **end if** 12  **end for** 13  Save trained encoder *f_en_c*(·) 14  **for each** *x* ∈ 𝒟 **do** 15    *z* ← *f_en_c*(*x*)     // latent vector of dimension *d* 16    Store *z* for fuzzy classification 17  **end for**

## 3. Architecture and Learning Mechanism of the Fuzzy Neural Network in FAIR-Net

### 3.1. Fuzzy Rule-Based Network Structure with FCM Clustering

The fuzzy neural network (FNN) component of FAIR-Net is designed to provide interpretable and robust classification for ancient Chinese character recognition [25]. Inspired by the architecture of radial basis function neural networks (RBFNNs), our model replaces the hidden layer’s conventional activation functions with fuzzy rules derived from Fuzzy C-Means (FCM) clustering, enabling semantic interpretability of each decision path.

The FNN consists of three main layers:

- Input layer: Receives the d-dimensional latent feature vectors (*d* ∈ {10, 20, 30, 40}) from the autoencoder.
- Hidden layer: Composed of fuzzy rule nodes generated via FCM clustering on the encoded feature space.
- Output layer: Performs linear aggregation followed by a Softmax transformation to produce probabilistic predictions over character classes.

Each hidden node corresponds to a fuzzy rule of the following form:

If *x* is *u*_i_(*z*), Then *g*_i_(*z*) = *a*_i0_ + *a*_i1_*z*_1_ + … + *a*_in_ *z*_n_. (1)

here, *u*_i_(*z*) denotes the fuzzy membership of input *x* to the *i*-th cluster, and *g*_i_(*x*) is a linear function representing the rule’s consequent.

**Figure 2:**
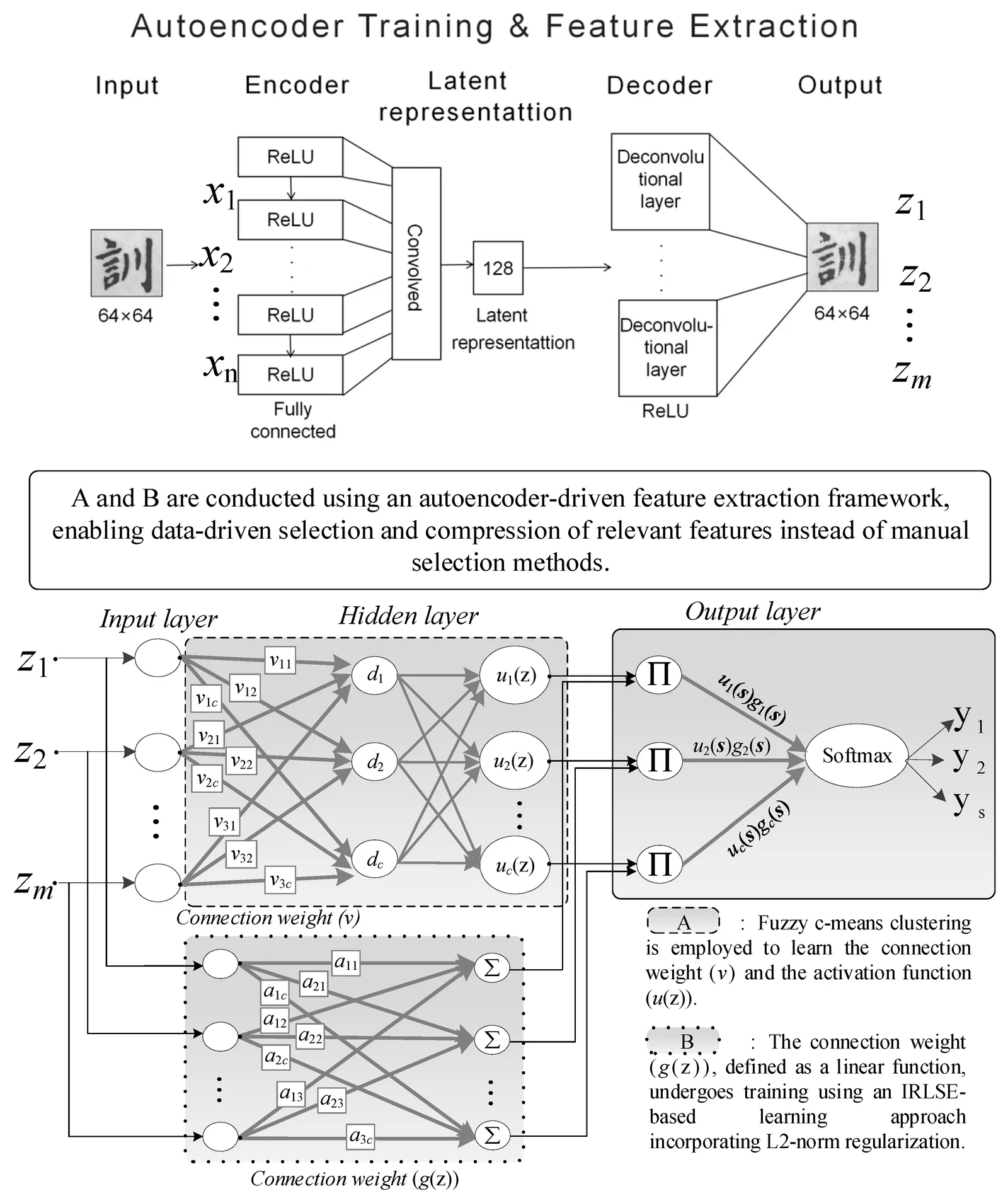
Overall network architecture framework including inherent algorithmic details of the FAIR-Net.

As shown in Figure 2, to establish the fuzzy rule nodes in the hidden layer of the proposed network, we apply Fuzzy C-Means (FCM) clustering on the encoded low-dimensional character features extracted by the autoencoder. Each cluster corresponds to one fuzzy rule node, and the cluster centers serve as the antecedent parameters of the fuzzy rules.

The connection weights between the input vector and the hidden layer nodes are initialized based on the cluster centers obtained from FCM. Specifically, for the i-th fuzzy node (corresponding to the *i*-th cluster), the center vector $v_{i}\in {\mathbb{R}}^{d}$ is computed as:

(2) $$v_{i}=\frac{\sum_{j-1}^{N}u_{ij}^{m}z_{j}}{\sum_{j-1}^{N}u_{ij}^{m}}$$

where, $z_{j}$ is the *j*-th input sample in the training set, $u_{ij}$ ∈ [0, 1] is the membership degree of sample $z_{j}$ to cluster *i*, *m* > 1 is the fuzzification coefficient, *N* is the total number of training samples.

To better capture shape variation and degradation in ancient characters, we employ a custom distance metric that considers intra-cluster dispersion without requiring full covariance normalization (unlike Mahalanobis distance). The distance between input *x* and cluster center $v_{i}$ is defined as:

(3) $$d_{i}(z)=\frac{∥z-v_{i}{∥}^{2}}{\sigma_{i}^{2}}$$

where, $\sigma_{i}^{2}$ denotes the variance of samples within cluster *i*. This distance formulation enhances numerical stability and reduces the risk of overfitting caused by noise correlations across features.

The membership degree of input *x* to the *i*-th fuzzy cluster is then computed as:

(4) $$u_{i}(z)={\left(\sum_{k=1}^{c}{\left(\frac{d_{i}(z)}{d_{k}(z)}\right)}^{\frac{2}{m-1}}\right)}^{-1}$$

where, *c* is the number of clusters (i.e., fuzzy rules). The resulting $u_{i}(z)$ is used as the activation value of the *i*-th hidden node.

To overcome ambiguity in membership-based activations—especially in cases where visually different characters produce similar distances—we design the consequent part of each fuzzy rule as a linear function of the input vector:

(5) $$g_{i}(z)=a_{i0}+a_{i1}z_{1}+a_{i2}z_{2}+\cdots +a_{id}z_{d}=a_{i}^{T}z$$

where, $a_{i}={\left[a_{i0},a_{i1},\ldots ,a_{id}\right]}^{T}$ is the coefficient vector of the *i*-th rule, *d* is the input feature dimension (in this case, directly set to *d* ∈ {10, 20, 30, 40} after autoencoding). These coefficients $a_{i}$ are optimized using Iteratively Reweighted Least Squares Estimation (IRLSE) on the training data.

The final class score for class *s* is computed by aggregating all rule outputs (i.e., hidden layer node outputs), each weighted by its fuzzy membership degree:

(6) $$f_{s}(z)=\sum_{i=1}^{c}u_{i}(z)\cdot g_{i}^{s}(z)$$

where $g_{i}^{s}(zx)$ denotes the linear output of the *i*-th fuzzy rule for class *s*. This rule-based aggregation allows the model to express complex, nonlinear boundaries in a locally linear and interpretable form (6).

### 3.2. Newton’s Method-Based IRLS for Output Layer Parameter Estimations

To convert the class scores $f_{s}(z)$ into a probabilistic output, the output layer is designed in two stages: score normalization and weight optimization. The raw class scores are passed through a Softmax function to yield normalized class probabilities:

(7) $${\hat{y}}_{s}(z)=\frac{\exp(f_{s}(z))}{\sum_{j=1}^{S}\exp(f_{j}(z))}$$

where, ${\hat{y}}_{s}(z)$ ∈ [0, 1] represents the predicted probability of input *x* belonging to class *s*, and *S* is the total number of character classes (e.g., 3755 for CompetitionDB). This two-stage fuzzy logic–inspired process enables the model to perform robust multi-class classification while maintaining interpretability of its internal decision rules—an important requirement for ancient script recognition where ground truth may be uncertain or controversial.

The final predicted label is assigned based on maximum likelihood:

(8) $$\hat{s}=\arg\underset{s}{\max}{\hat{y}}_{s}(z)$$

To optimize the output layer weights, we use a standard cross-entropy (CE) loss function combined with *ℓ2* regularization, which mitigates overfitting:

(9) $$L=-\sum_{i=1}^{N}\sum_{s=1}^{S}r_{i}^{s}\log{\hat{y}}_{i}^{s}+\frac{\lambda }{2}\sum_{s=1}^{S}∥W_{s}{∥}^{2}$$

where, $r_{i}^{s}$, ∈ {0, 1} is the one-hot target label for class *s*, ${\hat{y}}_{i}^{s}$ is the predicted probability for class *s*, $W_{s}\in {\mathbb{R}}^{d+1}$ contains the output layer weights (including bias), *λ* is a regularization coefficient.

Since the CE loss does not allow a closed-form solution, we adopt an IRLS optimization strategy based on Newton’s method. For each class *s*, the weight vector is updated at iteration *g* as:

(10) $$W_{s}^{\left(g+1\right)}={\left({\tilde{Z}}^{⊤}{\tilde{D}}_{s}\tilde{Z}+\lambda I\right)}^{-1}{\tilde{Z}}^{⊤}{\tilde{D}}_{s}{\tilde{r}}_{s}$$

Here,

$$\tilde{Z}=\left[\begin{array}{cccc} \mu_{11}z_{11} & \ldots & \mu_{1c}z_{1d} & \mu_{1c}\\ \mu_{21}z_{21} & \ldots & \mu_{2c}z_{2d} & \mu_{2c}\\ \vdots & \ddots & \vdots & \vdots \\ \mu_{N1}z_{N1} & \ldots & \mu_{Nc}z_{Nd} & \mu_{Nc} \end{array}\right],\;R_{s}=\left[\begin{array}{c} r_{1}^{s}\\ r_{2}^{s}\\ \vdots \\ r_{N}^{s} \end{array}\right]\;\mathrm{with}\;r_{i}^{s}\in \{0,1\}$$

$$\tilde{D}=\left[\begin{array}{cccc} {\hat{y}}_{1}(1-{\hat{y}}_{1}) & 0 & \ldots & 0\\ 0 & {\hat{y}}_{2}(1-{\hat{y}}_{2}) & \ldots & 0\\ \vdots & \vdots & \ddots & \vdots \\ 0 & 0 & \ldots & {\hat{y}}_{N}(1-{\hat{y}}_{N}) \end{array}\right]$$

$$W_{s}^{\left(g\right)}={\left[w_{11}^{\left(g\right)}\;\ldots \;w_{1(d+1)}^{\left(g\right)}\;\ldots \;w_{c(d+1)}^{\left(g\right)}\right]}^{⊤}$$

## 4. Design Framework of the Proposed Fuzzy Neural Recognition System

**Figure 3:**
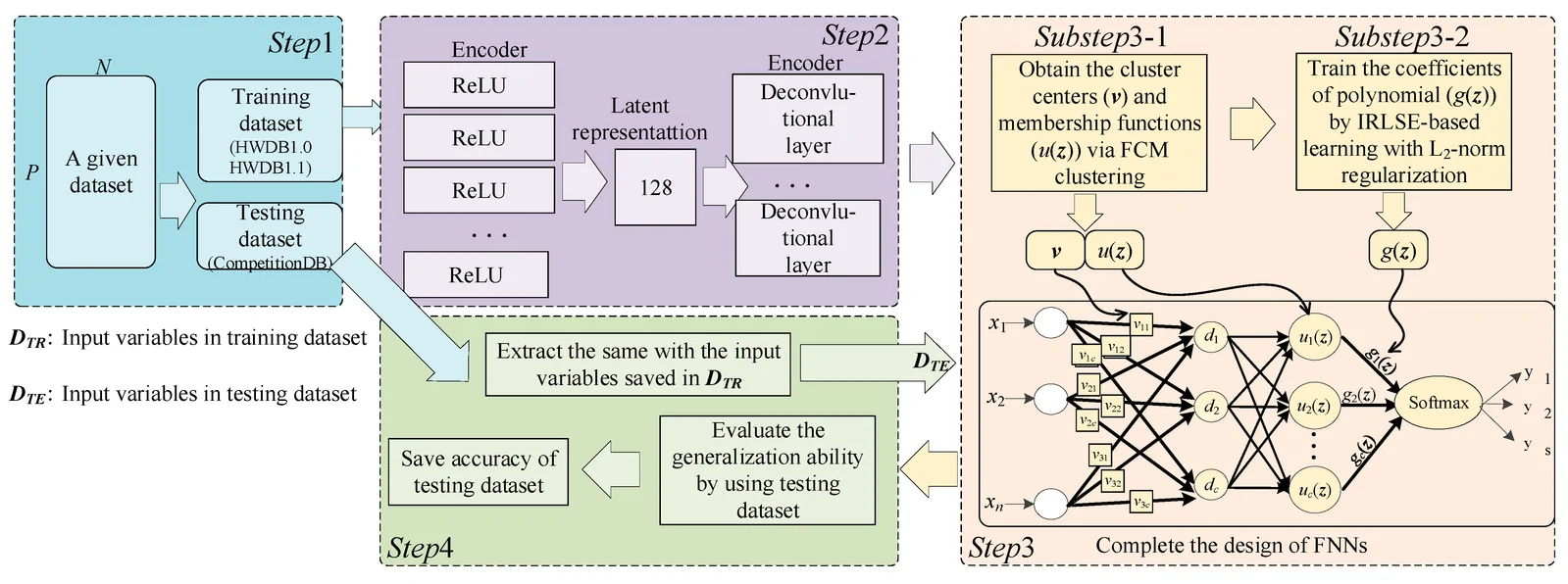
Overall Network Architecture of FAIR-Net.

This section presents the complete design workflow of FAIR-Net, our interpretable and modular fuzzy neural recognition system for ancient Chinese character classification. The framework is composed of three main stages: feature extraction, fuzzy rule induction, and interpretable probabilistic classification. An overview of the system architecture and data flow is illustrated in Figure 3.

Step 1: Data Preparation and Experimental Partitioning

The dataset is divided into training and testing subsets using stratified k-fold cross-validation to ensure balanced character distribution across folds.

- Preprocessing: All character images are converted to grayscale, resized to 64 × 64, and normalized to [0, 1]. Standard data augmentation strategies (random rotation, translation, Gaussian noise) are optionally applied to improve robustness.
- Partitioning: The training set is used for autoencoder training, hyperparameter tuning, and fuzzy classifier learning, while the testing set is reserved exclusively for evaluating generalization performance.

Step 2: Feature Extraction via Autoencoder

To reduce dimensionality and suppress noise, a deep autoencoder is trained in an unsupervised manner:

- Encoder: Three convolutional layers followed by two fully connected layers map the input image x ∈ ℝ^64×64^ to a latent vector z ∈ ℝ*^d^*, where *d* ∈ {10, 20, 30, 40}.
- Decoder: A mirrored structure reconstructs the input image from the latent representation, ensuring that the latent features preserve semantic structure.
- Training Objective: The autoencoder is optimized using Mean Squared Error (MSE) loss with Adam optimizer. Early stopping is employed to prevent overfitting.

The final encoder function *f_en_c(·)* maps each input image to a compact latent vector *z*, which serves as the input for the fuzzy neural classifier in Step 3.

Step 3: Construction of the Fuzzy Neural Classifier

The learning process for the fuzzy neural network is performed in two sequential stages: one focusing on fuzzy rule induction, and the other on class-specific decision mapping.

Substep 3.1: Learning the Hidden Layer via FCM Clustering

- The hidden layer is constructed using Fuzzy C-Means (FCM) clustering applied to the latent feature vectors (**z**) obtained from the autoencoder.
- Each cluster center v_i_ defines the premise of a fuzzy rule, while the corresponding fuzzy membership function *μ*_i_(**z**), computed directly from the FCM procedure, serves as the activation of the *i*-th hidden node.
- These membership activations quantify the degree to which an input vector belongs to each fuzzy prototype.
- Consequently, the fuzzy rule base provides an interpretable representation, where each rule corresponds to a localized region of the latent feature space.

Substep 3.2: Learning Output Weights via IRLS with L2 Regularization

Once the fuzzy rules are established, the connection weights between the hidden and output layers are learned using the Iteratively Reweighted Least Squares (IRLS) algorithm.

- A Cross-Entropy (CE) loss is used to train a multi-class softmax classifier over the fuzzy rule activations.
- To prevent overfitting and mitigate issues from feature collinearity, L2-norm regularization is added.
- This step ensures that the final decision layer maintains a balance between model expressiveness and numerical stability.

## 5. Experiments and Analysis

### 5.1. Dataset Description and Experimental Setup

To comprehensively evaluate the proposed FAIR-Net framework across both modern handwritten and ancient script recognition scenarios, we employ four datasets:

- CASIA-HWDB1.0: Contains 3740 common Chinese characters based on GB2312-80 level-1 standard, written by 420 different individuals.
- CASIA-HWDB1.1: Covers 3755 character classes written by 300 new individuals.
- ICDAR2013 CompetitionDB: A test set of 3755 characters contributed by another 60 writers, officially used in the ICDAR 2013 offline Chinese handwriting recognition competition.
- Ancient Chinese Character Dataset: A large-scale, self-constructed dataset covering 9233 distinct classes of ancient Chinese scripts (oracle bone inscriptions, bronze inscriptions, large/small seal scripts, bamboo and silk manuscripts, coin inscriptions, and stone carvings). The dataset contains 979,907 images after enhancement (originally 673,639), sourced from scanned archaeological rubbings and photographs.

As shown in Table 1, for CASIA-HWDB1.0/1.1 and ICDAR2013 CompetitionDB, characters are preprocessed into grayscale 64 × 64 images and normalized to the [0, 1] range. Following prior work, CASIA-HWDB1.0 and CASIA-HWDB1.1 are used jointly for training and validation under a five-fold cross-validation protocol, ensuring stable reporting of performance. After model selection, final evaluation is conducted on CompetitionDB as a fixed held-out test set. This split is consistent with the official ICDAR benchmark and previous literature, avoiding protocol mismatch.

For the Ancient Chinese Character Dataset, images are preprocessed by median filtering to remove salt-and-pepper noise, standardized to 256 × 256 pixels while preserving aspect ratio, and augmented using a two-step adaptive enhancement and resampling strategy to alleviate the long-tail distribution. The dataset is split approximately 4:1 into training and testing sets.

As shown in Table 2, we compare FAIR-Net with two groups of baselines: Modern handwriting OCR baselines—five CNN-based models widely used in handwritten Chinese character recognition: AlexNet [27], GoogLeNet [28], CNN-Fujitsu [28], ART-CNN [29], and R-CNN Voting [19]. Ancient script recognition baselines—state-of-the-art CNN and Vision Transformer backbones evaluated on the Ancient Chinese Character Dataset including ResNet101 [14], SwinT-v2-small [15].

The fuzzy classifier in FAIR-Net is trained with variable settings of feature dimensionality (10, 20, 30, 40), cluster number (2–5), and regularization parameters (Table 3).

All experiments were conducted on a workstation equipped with an Intel® Xeon® Gold 5218 CPU (Intel Corporation, Santa Clara, CA, USA) and four RTX A6000 48-GB GPUs (NVIDIA Corporation, Santa Clara, CA, USA), hosted at the China–Korea Big Data and Artificial Intelligence Research Center. For FAIR-Net, the reported “25 s” refers to the inference time for the full test set under batch size 128. For ResNet101 and SwinT-v2-small, the reference time corresponds to ~53.6 ms per image under batch size 32, as reported in prior work.

### 5.2. Experimental Results and Comparative Evaluation (CompetitionDB)

To investigate the internal robustness and flexibility of FAIR-Net, we conducted extensive experiments by varying two key hyperparameters: the feature dimensionality of the autoencoder’s latent space (10, 20, 30, and 40), and the number of clusters used in the fuzzy classifier (2, 3, 4, and 5). The results are summarized in Table 4, which reports both training and testing accuracy (mean ± standard deviation). Overall, the model demonstrates strong performance across all configurations, with an average training accuracy of 98.52% and testing accuracy of 96.96%, reflecting FAIR-Net’s stable generalization capabilities.

Among all settings, the best result (97.91% ± 1.03%) was achieved when using 10-dimensional features with 3 clusters, demonstrating that even highly compressed representations can preserve the discriminative semantics of ancient character structures. Performance remains comparably strong with 20- and 40-dimensional features when paired with 3 clusters, further validating the autoencoder’s ability to extract robust low-dimensional embeddings. In contrast, using fewer clusters (e.g., 2) tends to underfit the decision space, while more clusters (e.g., 4 or 5) may lead to overfitting or unstable membership boundaries, as indicated by the higher variance. These findings suggest that a moderate cluster count (k = 3) is optimal for capturing structural ambiguity without introducing excessive rule complexity.

From the cluster-wise comparison, it is evident that the number of fuzzy clusters plays a critical role in shaping classification boundaries. Using 3 clusters consistently yielded the highest or near-highest accuracy across all feature dimensions, suggesting it offers the best balance between expressiveness and generalization. Notably, increasing the number of clusters beyond 3 does not result in better accuracy—in some cases, it even introduces performance degradation. This outcome aligns with fuzzy clustering theory, where over-clustering can fragment decision regions and amplify uncertainty. On the other hand, excessively low cluster counts limit the model’s capacity to represent nuanced glyph variations, especially in stylistically diverse or partially eroded samples.

Table 5 presents a comparative analysis of the proposed FAIR-Net against five representative baseline models in terms of testing accuracy and inference time. FAIR-Net achieves a testing accuracy of 97.91% ± 1.03% and an inference time of 25 s per batch. At the same time, models such as GoogLeNet (96.26% ± 1.18%) also yield competitive accuracy, though with relatively higher computational time. CNN-Fujitsu, despite its more complex architecture, shows relatively lower performance in both accuracy and efficiency.

**Figure 4:**
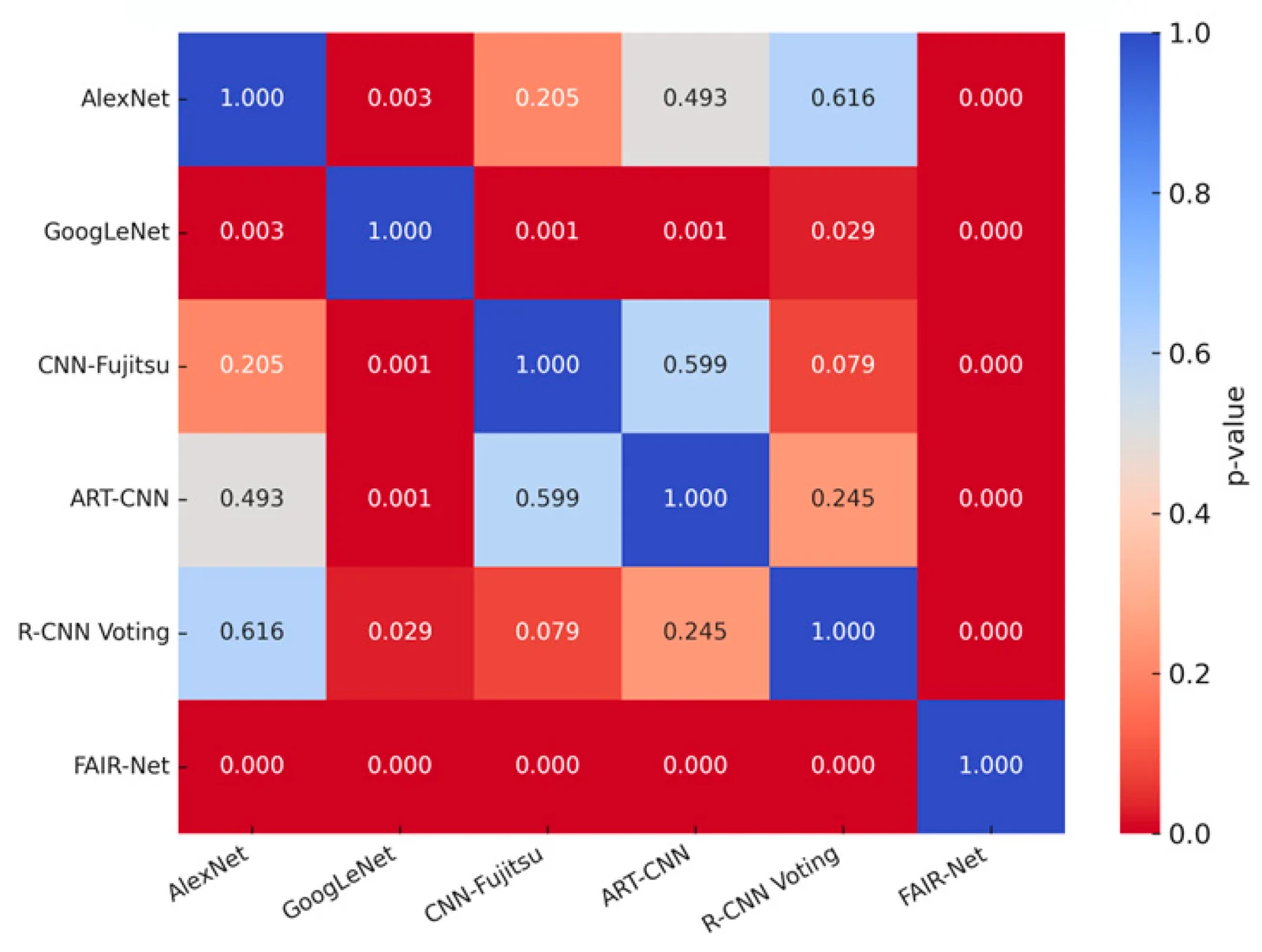
Heatmap of paired *t*-test results between FAIR-Net and baseline models on CompetitionDB.

To further ensure that these differences are statistically sound, we conducted paired *t*-tests between FAIR-Net and all baseline models. The results (*p* < 0.001 across all comparisons) confirm that the observed accuracy gains are statistically significant. For better clarity, Figure 4 presents a heatmap visualization of the pairwise significance results, where warmer or redder colors correspond to smaller *p*-values. This visualization helps readers intuitively assess the robustness of the comparisons. It should also be noted that the reported results for baseline models were taken from prior literature, and slight differences in implementation settings (e.g., training epochs, learning rate schedules, or augmentation strategies) could influence the absolute performance. Therefore, the comparisons mainly serve as representative references rather than strict apples-to-apples benchmarks.

Overall, while FAIR-Net shows promising improvements in both accuracy and computational efficiency under the current evaluation settings, we would like to emphasize that these results should be interpreted with caution. The comparison relies on reported baseline configurations from prior literature, and variations in implementation details (e.g., training epochs, learning rate schedules, data augmentation) may influence the absolute performance. Therefore, our main contribution lies not in claiming absolute superiority, but in demonstrating that FAIR-Net can achieve a favorable trade-off between recognition accuracy, interpretability, and efficiency.

### 5.3. Ancient Chinese Character Dataset Evaluation

To further examine the adaptability of FAIR-Net beyond modern handwritten Chinese characters, we evaluated it on the Ancient Chinese Character Dataset (9233 classes; 979,907 samples after enhancement). The dataset covers diverse script styles, including oracle bone inscriptions, bronze inscriptions, large and small seal scripts, bamboo and silk manuscripts, coin inscriptions, and stone carvings. All models were trained from scratch under identical preprocessing (median filtering, resizing to 256 × 256, normalization to [0, 1]) and data augmentation settings, with a 4:1 train/test split.

From Table 6, FAIR-Net achieves a testing accuracy of 83.25% ± 0.48%, which is slightly higher than ResNet101 but falls short of SwinT-v2-small by 2.49%. In terms of training accuracy, FAIR-Net also lags behind both baselines. This performance gap suggests that while FAIR-Net’s compact latent representation and fuzzy-rule classifier are effective for modern handwritten data, they may have limitations in modeling the highly complex and stylistically diverse glyph structures of ancient scripts, particularly under extreme class imbalance. Despite this, FAIR-Net maintains a substantial efficiency advantage—its total training time of 4 h 24 m is 5.5× faster than SwinT-v2-small and 6.5× faster than ResNet101 under identical hardware and settings. This efficiency stems from its lightweight convolutional encoder and reduced parameter count, making it suitable for scenarios where rapid model iteration or deployment on resource-constrained environments is prioritized over peak accuracy.

**Figure 5:**
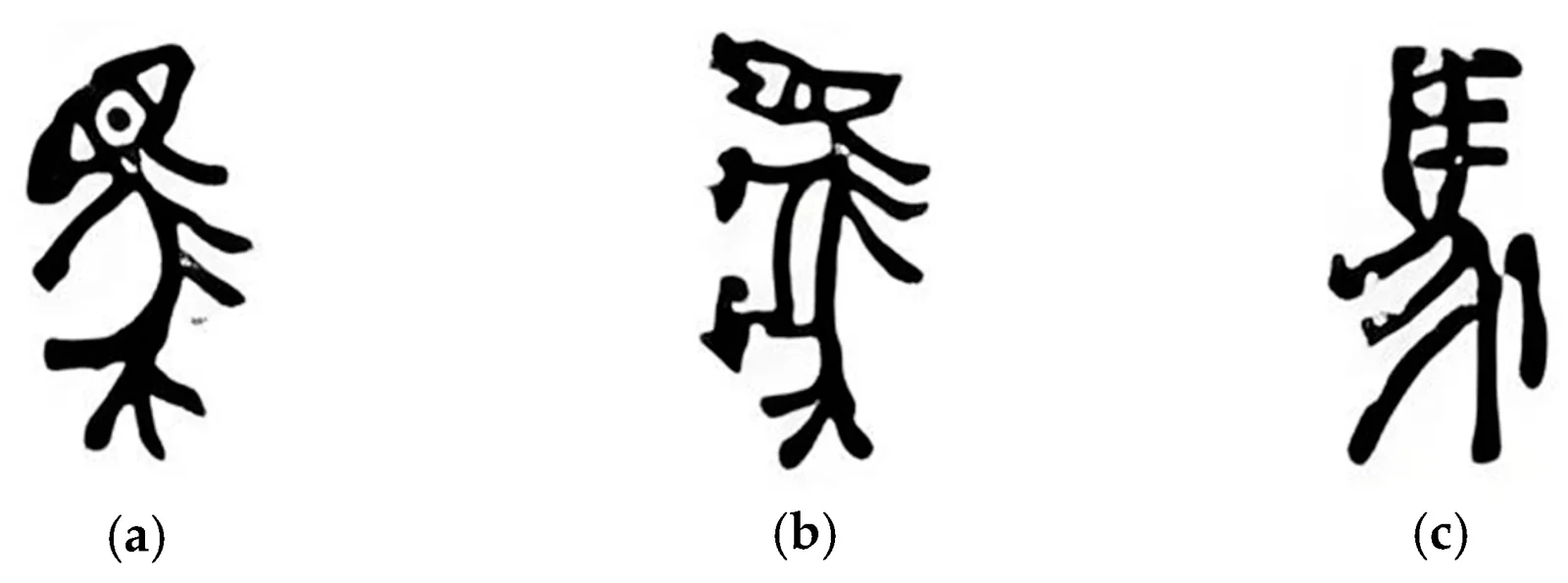
Examples of the character “horse” across three ancient scripts: (**a**) Oracle Bone Script (OBS), (**b**) Bronze Inscriptions (BI), and (**c**) Small Seal Script (SSS).

To further illustrate the interpretability of FAIR-Net, Figure 5 presents the evolution of the character “horse” in three representative script categories: Oracle Bone Script, Bronze Inscriptions, and Small Seal Script. Despite sharing the same semantic meaning, these scripts exhibit substantial stylistic differences—Oracle Bone Script retains a pictographic resemblance to a horse body, Bronze Inscriptions simplify the structure with heavier strokes, and Small Seal Script regularizes the glyph into a symmetrical geometric form. FAIR-Net effectively captures the shared semantic core of the character while simultaneously recognizing stylistic variations across different scripts. This demonstrates that the proposed framework not only achieves accurate classification but also provides semantic transparency, thereby offering a meaningful tool for analyzing the cultural evolution of ancient Chinese scripts.

The fuzzy rule representation in FAIR-Net further reinforces interpretability by expressing decision boundaries in explicit IF–THEN form, enabling human-readable tracing of how compact latent features from the autoencoder are mapped to final class decisions. Unlike conventional deep neural networks, which typically operate as opaque “black boxes”, FAIR-Net’s fuzzy rule layer provides semantic insight into the learned classification logic. To illustrate this mechanism, we present an example from the Ancient Chinese Character Dataset, focusing on three representative script categories: Oracle Bone Script (OBS), Bronze Inscriptions (BI), and Small Seal Script (SSS). Each input sample is represented by four selected latent features obtained from the encoder output after dimensionality reduction. The fuzzy rules for each class are as follows:

Class 1: “Oracle Bone Script (OBS)”

$$FR_{1}^{1}:\;\mathrm{If}\;z\;\mathrm{is}\;u^{\left(1\right)}\left(z\right),\;\mathrm{then}\;h_{1}^{\left(OBS\right)}=0.842+0.063z_{1}-0.048z_{2}+0.051z_{3}-0.039z_{4}$$

$$FR_{2}^{1}:\;\mathrm{If}\;z\;\mathrm{is}\;u^{\left(2\right)}\left(z\right),\;\mathrm{then}\;h_{2}^{\left(OBS\right)}=0.839+0.056z_{1}-0.052z_{2}+0.048z_{3}-0.041z_{4}$$

$$FR_{3}^{1}:\;\mathrm{If}\;z\;\mathrm{is}\;u^{\left(3\right)}\left(z\right),\;\mathrm{then}\;h_{3}^{\left(OBS\right)}=0.845+0.061z_{1}-0.049z_{2}+0.053z_{3}-0.038z_{4}$$

Class 2: “Bronze Inscriptions (BI)”

$$FR_{1}^{2}:\;\mathrm{If}\;z\;\mathrm{is}\;u^{\left(1\right)}\left(z\right),\;\mathrm{then}\;h_{1}^{\left(BI\right)}=0.624-0.072z_{1}+0.058z_{2}-0.047z_{3}+0.036z_{4}$$

$$FR_{2}^{2}:\;\mathrm{If}\;z\;\mathrm{is}\;u^{\left(2\right)}\left(z\right),\;\mathrm{then}\;h_{2}^{\left(BI\right)}=0.619-0.075z_{1}+0.061z_{2}-0.048z_{3}+0.034z_{4}$$

$$FR_{3}^{2}:\;\mathrm{If}\;z\;\mathrm{is}\;u^{\left(3\right)}\left(z\right),\;\mathrm{then}\;{\;h}_{3}^{\left(BI\right)}=0.628-0.069z_{1}+0.057z_{2}-0.046z_{3}+0.037z_{4}$$

Class 3: “Small Seal Script (SSS)”

$$FR_{1}^{3}:\;\mathrm{If}\;z\;\mathrm{is}\;u^{\left(1\right)}\left(z\right),\;\mathrm{then}\;\;h_{1}^{\left(SSS\right)}=0.873-0.067z_{1}+0.053z_{2}-0.050z_{3}+0.042z_{4}$$

$$FR_{2}^{3}:\;\mathrm{If}\;z\;\mathrm{is}\;u^{\left(2\right)}\left(z\right),\;\mathrm{then}\;\;h_{2}^{\left(SSS\right)}=0.868-0.071z_{1}+0.038z_{2}-0.053z_{3}+0.050z_{4}$$

$$FR_{3}^{3}:\;\mathrm{If}\;z\;\mathrm{is}\;u^{\left(3\right)}\left(z\right),\;\mathrm{then}\;\;h_{3}^{\left(SSS\right)}=0.873-0.042z_{1}+0.061z_{2}-0.056z_{3}+0.058z_{4}$$

These fuzzy rules delineate distinct regions in the latent feature space with corresponding linear consequents, thereby rendering the decision-making process transparent. The variations in coefficients across rules within the same class capture intra-class diversity, such as stylistic differences observed between different historical stages of Oracle Bone Script or Bronze Inscriptions. Moreover, the sign and magnitude of the coefficients indicate the directional contributions of individual latent features to classification confidence, providing semantic insight into which structural or stroke-level characteristics most strongly influence the final decision.

### 5.4. Ablation Study

To investigate the individual contribution of each component in the fuzzy classification module of FAIR-Net, we conducted ablation experiments under four different feature dimensionalities (10, 20, 30, 40). The results are summarized in Table 7, where we compare three variants of the fuzzy classifier:

- FCM-LSE: A basic model using Fuzzy C-Means clustering with Least Squares Estimation as the output layer, without any robust loss or regularization.
- FCM-IRLS-CE: A stronger variant incorporating Iteratively Reweighted Least Squares (IRLS) and the Cross Entropy (CE) loss, improving robustness to noisy labels and outliers.
- FCM-IRLS-CE-L2 (Proposed): The full version of our classifier, adding L2 regularization to further stabilize learning and prevent overfitting.

As shown in Table 7, the full FAIR-Net model (FCM-IRLS-CE-L2) consistently achieves the best performance across both datasets and all feature dimensionalities. On ICDAR2013 CompetitionDB, it achieves a peak accuracy of 97.91% ± 1.03%, outperforming the other variants while also reducing variance, which indicates more stable learning. On the Ancient Chinese Character Dataset, although the overall accuracy is lower due to its greater class diversity and stylistic complexity, the proposed model still surpasses the alternatives, reaching 83.25% ± 1.48% at 20-dimensional features.

Comparing the variants, FCM-LSE performs the weakest, with the lowest accuracy and highest variance, highlighting its vulnerability to outliers. Adding IRLS-CE yields clear gains (e.g., +1.4% on the ancient dataset with 10 features), demonstrating the effectiveness of robust optimization and loss design. The final addition of L2 regularization provides further but smaller improvements (~0.2–0.3%), while consistently lowering variance. These results confirm that each module—IRLS optimization, CE loss, and L2 regularization—plays a complementary role. The ablation validates the theoretical design of FAIR-Net’s fuzzy inference module and demonstrates its robustness and stability across both standard and challenging ancient-script datasets.

## 6. Conclusions

In this paper, we proposed FAIR-Net, a novel hybrid architecture that integrates deep unsupervised representation learning with interpretable fuzzy rule-based classification for robust recognition of ancient Chinese characters. The model is designed to address the core challenges in historical script digitization—including severe degradation, stylistic diversity, and the scarcity of large-scale labeled data—by combining a deep autoencoder for compact, noise-resistant feature extraction with a Fuzzy Neural Network (FNN) that models soft semantic boundaries through Fuzzy C-Means clustering and rule inference.

Extensive experiments on three widely used offline handwritten Chinese character datasets (CASIA-HWDB1.0, CASIA-HWDB1.1, and ICDAR2013 CompetitionDB) demonstrate that FAIR-Net achieves strong recognition performance while maintaining high computational efficiency. The model reaches a peak testing accuracy of 97.91% ± 1.03%, outperforming all CNN-based baselines with statistical significance (*p* < 0.01, Cohen’s d > 0.8). Ablation studies further validate the contribution of each component—IRLS optimization, CE loss, and L2 regularization—showing that their integration is necessary for stable and robust performance.

To further evaluate its applicability beyond modern handwriting, we additionally introduced a large-scale Ancient Chinese Character Dataset comprising over 9000 classes across multiple script types (oracle bone, bronze, seal, bamboo/silk, coin, and stone inscriptions). On this dataset, FAIR-Net achieved competitive recognition performance while requiring significantly lower training and inference costs compared to deep CNNs and Vision Transformer baselines (e.g., ResNet101, Swin Transformer). Although its accuracy is slightly lower than the strongest transformer-based models, FAIR-Net offers substantial efficiency gains and, importantly, provides interpretable decision rules that reveal semantic insights into how ancient glyphs are classified.

In summary, FAIR-Net not only delivers strong empirical results on both modern handwritten and ancient script datasets but also contributes interpretability through its fuzzy rule-based reasoning, which is particularly valuable in the domain of historical script analysis. For future work, we plan to extend FAIR-Net to multilingual ancient scripts, explore integration with self-supervised contrastive learning, and investigate adaptive fuzzy rule bases to further enhance its generalization to extremely low-resource and stylistically diverse scenarios. We acknowledge that throughput comparisons under identical resolutions and batch sizes would provide a more standardized evaluation; this will be addressed in future work.

## Figures and Tables

**Table 1:** Overview of Handwritten Chinese Character Datasets.

Dataset	Sample Size	Image Dimension	Number of Classes	Number of Writers	Usage
CASIA-HWDB1.0	1,680,258	64 × 64 (raw)	3740	420	Training/Validation
CASIA-HWDB1.1	1,172,907	64 × 64 (raw)	3755	300	Training/Validation
ICDAR2013 CompetitionDB	224,000	64 × 64 (raw)	3755	60	Testing
Ancient Chinese Character Dataset	979,907 (enhanced; originally 673,639)	256 × 256 (raw)	9233	N/A	Training/Testing

**Table 2:** Architectural Settings and Preprocessing Parameters of Evaluated Models.

Model	Input Size	Mask Size	Normalization	Convolution Layers	Fully Connected Layers
AlexNet	108 × 108	114 × 114	[0, 1] float	5	2
GoogLeNet	112 × 112	120 × 120	[0, 1] float	22	1
CNN-Fujitsu	96 × 96	120 × 120	[0, 1] float	3	2
ART-CNN	96 × 96	120 × 120	[0, 1] float	7	2
R-CNN Voting	96 × 96	120 × 120	[0, 1] float	7	2
ResNet101	224 × 224/256 × 256	–	[0, 1] float	101	1
SwinT-v2-small	256 × 256	7	[0, 1] float	–	2
FAIR-Net (Proposed)	Latent vector (10–40)	–	–	–	–

Note: Input resolutions vary across models (96–112 px for CNNs, 224–256 px for ResNet/SwinT, and compact latent vectors for FAIR-Net). Thus, wall-clock timing comparisons should be interpreted with caution.

**Table 3:** Hyperparameter Space for the Proposed FAIR-Net.

Parameter	Range/Values
Number of Clusters	{2, 3, 4, 5}
Fuzzification Coefficient (m)	{1.1, 1.2, 2.0}
Burn-in Iterations	{50, 100, 150}
Thinning Interval	{25, 50, 75}
L2 Regularization Coefficient	{0.0001, 0.001, 0.01, 0.05}

**Table 4:** Performance of FAIR-Net with Varying Feature and Cluster Settings.

Number of Features	Number of Clusters	Training Accuracy (%)	Testing Accuracy (%)
10	2	98.30 ± 0.41	96.68 ± 1.24
	3	98.61 ± 0.38	**97.91 ± 1.03**
	4	98.44 ± 0.55	96.38 ± 2.07
	5	**98.72 ± 0.49**	97.03 ± 1.89
20	2	98.33 ± 0.37	96.59 ± 1.66
	3	98.69 ± 0.46	**97.86 ± 1.27**
	4	98.50 ± 0.52	96.21 ± 2.38
	5	**98.78 ± 0.43**	97.01 ± 1.94
30	2	98.28 ± 0.60	96.50 ± 2.13
	3	**98.66 ± 0.42**	96.89 ± 1.42
	4	98.47 ± 0.58	96.42 ± 2.26
	5	98.28 ± 0.48	**97.79 ± 1.12**
40	2	98.35 ± 0.39	96.62 ± 1.78
	3	98.67 ± 0.36	**97.83 ± 1.01**
	4	98.48 ± 0.57	96.47 ± 2.06
	5	**98.79 ± 0.41**	97.12 ± 1.95
Avg.		98.52	96.96

Mean ± std (%) over 5-fold experiments. Avg. indicates the average classification accuracy (best results are shown in bold).

**Table 5:** Performance Comparison of Baseline Models and FAIR-Net.

Algorithms	Testing Accuracy (%)	Computing time
AlexNet [27]	95.49 ± 1.22	43 s
GoogLeNet [28]	96.26 ± 1.18	2 m 24 s
CNN-Fujitsu [28]	94.77 ± 1.65	36 m 15 s
ART-CNN [29]	95.04 ± 1.47	1 m 16 s
R-CNN Voting [19]	95.55 ± 1.30	4 m 53 s
FAIR-Net (Proposed)	97.91 ± 1.03	25 s

**Table 6:** Performance Comparison on Ancient Chinese Character Dataset.

Algorithms	Training Accuracy (%)	Testing Accuracy (%)	Computing Time
ResNet101 [14]	85.42 ± 0.57	83.09 ± 1.64	28 h 36 m
SwinT-v2-small [15]	**87.16 ± 0.49**	**85.74 ± 2.52**	24 h 24 m
FAIR-Net (Proposed)	84.03 ± 0.46	83.25 ± 1.48	**4 h 24 m**

Mean ± std (%) over 5-fold experiments. Best results are in bold.

**Table 7:** Ablation studies of proposed FAIR-Net.

Data	Work	10 Features	20 Features	30 Features	40 Features
ICDAR2013 CompetitionDB	FCM-LSE	94.82 ± 1.94	94.51 ± 2.12	94.75 ± 1.87	94.43 ± 2.05
	FCM-IRLS-CE	97.52 ± 1.20	97.41 ± 1.36	97.33 ± 1.18	97.46 ± 1.15
	FCM-IRLS-CE-L2 (Proposed)	97.91 ± 1.03	97.86 ± 1.27	97.79 ± 1.12	97.83 ± 1.01
Ancient Chinese Character Dataset	FCM-LSE	81.47 ± 1.82	81.96 ± 1.77	81.88 ± 1.90	81.41 ± 1.98
	FCM-IRLS-CE	82.87 ± 1.55	82.96 ± 1.60	82.83 ± 1.62	82.90 ± 1.58
	FCM-IRLS-CE-L2 (Proposed)	82.98 ± 1.50	83.25 ± 1.48	82.94 ± 1.45	83.02 ± 1.53

## Data Availability

The original contributions presented in this study are included in the article. Further inquiries can be directed to the corresponding authors.
